# Natal colony influences age-specific movement patterns of the Yellow-legged gull (*Larus michahellis*)

**DOI:** 10.1186/s40462-023-00375-4

**Published:** 2023-02-11

**Authors:** Charly Souc, Nicolas Sadoul, Thomas Blanchon, Marion Vittecoq, Christophe Pin, Eric Vidal, Alain Mante, Rémi Choquet, Karen D. McCoy

**Affiliations:** 1 MIVEGEC, University of Montpellier, CNRS, IRD, Montpellier, France; 2 CEFE, University of Montpellier, CNRS, EPHE, IRD, University of Paul Valery Montpellier 3, Montpellier, France; 3 Present Address: Les Amis des Marais du Vigueirat, Marais du Vigueirat, Arles, France; 4 Tour du Valat, Research Institute for the Conservation of Mediterranean Wetlands, Arles, France; 5 Institut Mediterraneen de Biodiversite et d’Ecologie marine et continentale (IMBE), Aix Marseille Université, CNRS, IRD, Avignon Université, Aix-en-Provence, France; 6 Present Address: UMR Entropie, Labex-Corail, IRD, Noumea, New Caledonia; 7 Present Address: Parc national des Calanques, Marseille, France

**Keywords:** Capture heterogeneity, Colonial seabirds, Demography, Dispersal, Environmental quality, Migration, Multi-site mark-recapture, Prospection, Survival

## Abstract

**Background:**

As for other life history traits, variation occurs in movement patterns with important impacts on population demography and community interactions. Individuals can show variation in the extent of seasonal movement (or migration) or can change migratory routes among years. Internal factors, such as age or body condition, may strongly influence changes in movement patterns. Indeed, young individuals often tend to move across larger spatial scales compared to adults, but relatively few studies have investigated the proximate and ultimate factors driving such variation. This is particularly the case for seabirds in which the sub-adult period is long and difficult to follow. Here, we examine migration variation and the factors that affect it in a common Mediterranean seabird, the Yellow-legged gull (*Larus michahellis*).

**Methods:**

The data include the encounter histories of 5158 birds marked as fledglings between 1999 and 2004 at 14 different colonies in southern France and resighted over 10 years. Using a multi-event mark-recapture modeling framework, we use these data to estimate the probability of movement and survival, taking into account recapture heterogeneity and age.

**Results:**

In accordance with previous studies, we find that young individuals have greater mobility than older individuals. However, the spatial extent of juvenile movements depends on natal colony location, with a strong difference in the proportion of sedentary individuals among colonies less than 50 km apart. Colony quality or local population dynamics may explain these differences. Indeed, young birds from colonies with strong juvenile survival probabilities (~ 0.75) appear to be more sedentary than those from colonies with low survival probabilities (~ 0.36).

**Conclusions:**

This study shows the importance of studying individuals of different ages and from different colonies when trying to understand seabird movement strategies. Local breeding success and the availability of food resources may explain part of the among colony differences we observe and require explicit testing. We discuss our results with respect to the feedback loop that may occur between breeding success and mobility, and its potential implications for population demography and the dissemination of avian disease at different spatial scales.

**Supplementary Information:**

The online version contains supplementary material available at 10.1186/s40462-023-00375-4.

## Background

Among animals, seabirds are renowned for performing extensive movements, most of which are classified into one of three types: dispersal, migration or foraging. Dispersal is movement to a new location for reproduction, while migration represents cyclical movements between reproductive and non-reproductive periods [1]. Foraging occurs continuously and can largely constrain other types of movement [2, 3]. In addition to these three types of movements, young or failed breeders may also carry out prospecting behaviors, movements aimed to better evaluate future reproductive locations or find appropriate wintering areas [4–6]. All movements may be energetically costly for individuals, but these costs are generally offset by fitness gains in terms of survival and reproduction [7]. Some plasticity in dispersal behavior is known to occur [2, 8], allowing, for example, individuals to escape poor quality breeding habitats or a decline in local food resources. Plasticity may likewise occur in migratory movements, where individuals do not always migrate to the same extent or change migration patterns among years [9]. Internal factors, such as age, sex or body condition, may strongly influence these movement patterns. For example, young individuals often tend to show more widespread movement behavior compared to adults, differences that can be associated with prospecting activities [6, 10–12]. Seabirds take several years to reach sexual maturity (2–10 years) [13]. Little is known about seabird activities during this sub-adult period [14], but it can be of key importance for determining long-term fitness and population viability. Indeed, variation in vital rates of young age classes may strongly influence the overall metapopulation dynamics of a species and its evolutionary rate of change [15, 16]. A lack of experience during foraging can, for example, decrease survival rates of immatures [5]. Similarly, during prospecting young individuals may be exposed to diverse parasites and pathogens which may both modify their own survival and/or later reproductive success, and result in parasite dissemination to novel locations [1].

Seabird movement patterns can also be affected by anthropogenic activities that alter food sources and habitat availability. For example, it was shown that fishing practices, which facilitate foraging, likely contributed to changes in wintering areas used by Lesser black-backed gulls (*Larus fuscus*) [17]. Similarly, the presence of landfills was suggested to be directly related to body condition in juvenile Yellow-legged gulls [18]; the availability of such anthropogenic resources could alter both the motivation for birds to migrate or their ability to do so, depending on the relative quality and predictability of the resource [19, 20]. More generally, understanding the impact of environmental stresses on animal movement is still in its infancy and relatively few studies have investigated the proximate and ultimate factors influencing seabird movements.

Here, we examine variation in seasonal movement patterns in the Yellow-legged gull (*Larus michahellis*), an abundant species along the Mediterranean coast, with an opportunistic feeding ecology. The Yellow-legged gull (YLG) is frequently present in urbanized areas where it takes advantage of anthropogenic food sources [21]. However, despite its general pervasiveness, little is known about its movement patterns and the factors that influence them. This species has been divided into three subspecies—*atlantis, lusitanius, michahellis*. The *atlantis* subspecies is located only in part of Macaronesia, whereas the two other subspecies are present in Europe; *michahellis* is widely distributed between the Black Sea and the Atlantic coast, and *lusitanius* is confined to the northern Spanish coast. *Lusitanius* individuals are considered to be sedentary, with a majority of individuals remaining year-round within 50 km of their birthplace [22], regardless of their age and sex [23]. In contrast, the *michahellis* subspecies is considered as rather mobile. For example, young Algerian birds are sometimes seen in southern Europe during their first summer [24]. Similarly, young individuals of Adriatic populations can fly to distant areas [25], reaching as far as the Baltic Sea [26]. However, migratory distance seems to be lower in adult birds [25], a change that may be adaptive in many seabird species in order to arrive early at the breeding grounds and assure a high quality nest site [27]. As YLGs reach sexual maturity after 4 years of age, large-scale movements may decline gradually as individuals reach adulthood.

Colony-specific differences in migratory movements have also been observed among YLG populations [25, 26]. In the south of France, ringing data has suggested that juvenile gulls generally move north after fledgling in mid-summer. However, over-wintering areas may depend on the natal colony, with young from some colonies going towards the Alpine lakes and as far as the North Sea, and others using the Atlantic coast [28]. These initial observations were based on direct resightings only and did not quantify movement between distinct areas while taking into account individual variability, potentially caused by detection heterogeneities and age. They also did not address the proportion of non-migratory individuals in the different locations. However, these observations do raise the question of the possible drivers of colony-specific differences.

Here, we test for differences in migration strategies among individuals of different colonies, controlling for age (immature/adult). To do this, we applied a mark-recapture modeling approach to a large ringing dataset gathered from 14 YLG colonies of southern France in order to estimate movement probabilities between geographic zones while taking into account detection and survival probabilities. As outlined above, we expected young birds to be generally more mobile than adults due to prospecting behaviors and to life history constraints on adult movement. Based on previous observations, we also expected to find a colony effect on movement. In partial migratory species like YLGs, long distance movement may be a response to the quality of local environment [29], such as limited food resources, environmental stress and/or intra-specific competition at the breeding colony. Indeed, migration may lead to higher fitness when local conditions are poor both because resources may be more readily available elsewhere and because individuals can take advantage of this movement to evaluate potential breeding locations for future reproduction [8]. If conditions in the colony are good, year-round residency would be a better strategy since individuals do not incur the cost of migration [30]. Temporal variation in environmental quality is likely to select for plasticity in migration, with individuals moving in response to signals of environmental quality around the colony [7, 31]. Because the survival of young birds is directly related to the quality of the rearing environment [32], we used juvenile survival probabilities as a proxy for local conditions. We expected individuals from colonies where survival is low to move more frequently and further than those from colonies where survival is high.

## Methods

### Species and study site

The study area in the south of France is part of the distribution of the nominate subspecies *michahellis*, considered to be a partial migrant [33]. This subspecies is very common in its range, which includes a large part of the Mediterranean coast, the Black Sea and, since a recent expansion during the second half of twentieth century, the west coast and continental areas of France and other European countries. This expansion is likely explained by lower persecution by humans, combined with increased access to resources with the development of open-air dumps and fisheries offal [34]. In parallel to this expansion, YLGs have also begun to occupy urban centers and now breed in these areas in significant numbers [35, 36].

**Fig. 1:**
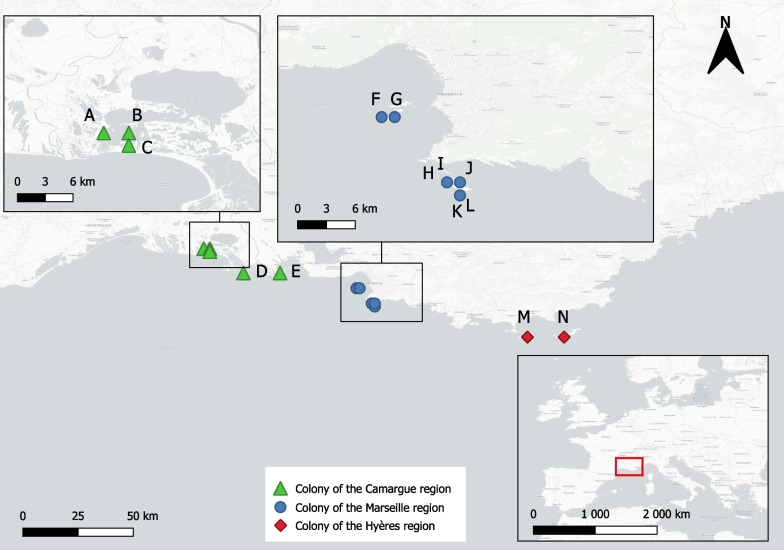
Colony locations. Localization of the studied colonies in France (inset) and their associated number of ringed individuals. *Camargue*: A—Besson (956), B—Flamants (733), C—Banaston (183), D—Galère (869), E—Pégoulier (396). *Marseille*: F—Pomègues (257), G—Ratonneau (16), H—Jarron (4), I—Jarre (112), J—Congloué (63), K—Riou (385), L—Plane (707). *Hyères*: M—Porquerolles (277), N—Bagaud (250)

Our dataset included 5158 individuals from 14 different colonies ringed as chicks between 1999 and 2004. These colonies lie in three main regions of south-eastern France: the Camargue, the Marseille archipelago, and the Hyères archipelago where 3137, 1544 and 477 chicks were ringed respectively (Fig. 1). All three regions are coastal with fishing ports and open-air dumps nearby. At capture, each bird was individually marked with a metal ring (MNHN, France) and a PVC ring stamped with an alpha-numeric character that can be read at distance. Amateur observers across Europe then observed these marked birds and transmitted the resighting information (ring number, date, geographic coordinates of the observation, age of the bird, behavior, etc*.*) to the coordinator of the ringing program. Ringing data was transmitted and centralized at the CRPBO, National Museum of Natural History, Paris, and then at EURING (European Union for Bird Ringing). These observations are therefore not standardized and can be qualified as opportunistic. In total, 3081 of the 5158 ringed individuals were resighted at least once between 1999 and 2011—the chosen period for data analyses.

### Mark recapture analysis

Resighting occasions, i.e. recaptures, were placed into 6-month capture periods that we termed seasons, based on the breeding phenology of the species. The period between May 15th and November 14th corresponds to the “post-breeding period”, while the period from November 15th to May 14th corresponds to the “pre-breeding period”. An individual was considered alive over a season if it was seen at least one during the time interval.

**Fig. 2:**
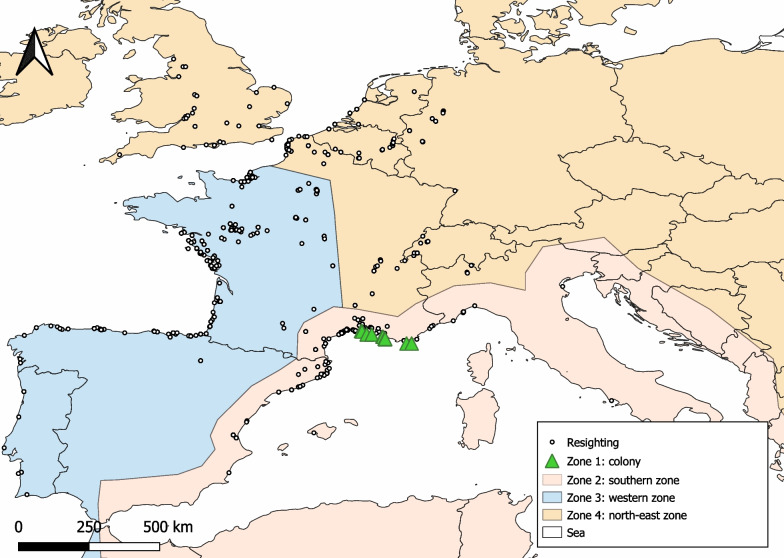
Geographic resighting zones. Map of the geographic sites considered for the mark-recapture model. Zone 1: less than 50 km around the natal colony. Open symbols indicate the resighting data of marked individuals used in analyses

We analyzed the data using a multi-event model [37], considering heterogeneous detection [38]. In particular, we quantified the probabilities of survival, recapture and transition between geographic zones, taking heterogeneous individual recapture rates into account. Four geographic zones were considered (Fig. 2): an enlarged natal zone (≤ 50 km around the natal colony) (zone 1), the southern zone (Mediterranean basin + 100 km inland) (zone 2), the western zone (Atlantic fringe along France and the Iberian Peninsula) (zone 3), and the north-east zone (rest of Europe, North Sea, Alps) (zone 4). The geographic limit between zones 3 and 4 was set to test the prediction that gulls from different natal regions exploit different areas—with eastern colonies (Hyères and Marseille) travelling further east (North Sea and Alps) than more western colonies (Camargue) [28]. When an individual was recorded in different geographic zones during the same season, an order of priority was established based on distance from the natal colony in order to determine its status; the furthest distance was considered as its state for the period. A bird was considered alive at its natal colony on its first capture (ringing), regardless of whether it was seen at other geographic locations or not during the following seasons.

We tested the goodness-of-fit of the Arnason-Schwarz [39] model (AS), which assumes no heterogeneity in survival (φ), transition (Ψ) or recapture (p) among individuals using the U-CARE program, version 2.3.4 [40]. Modeling was performed using E-Surge, version 2.2.3 [41]. For model selection, we applied the principle of parsimony using the AICc criterion (Aikake Information Criterion [corrected for small samples]) [42]. This criterion favors a sufficient number of parameters to fit the model to the data set, while being small enough to minimize parameter variance. Different effects were applied to the models. Among these, we included an effect of age with two classes: a juvenile class with individuals less than 6 months old, and an older class with individuals over 6 months old. Other types of age class divisions were tested, but were not retained in model selection (See Additional file 1: S1). In addition, effects of time, season, resighting zone and natal region (birthplace of the individuals) were included as parameters. We tested different natal region groupings to determine the scale at which parameters were homogeneous. After model selection, the parameters of interest and their 95% confidence intervals (CI) were estimated and interpreted.

## Results

Most resighting data came from coastal zones, but some inland areas also had high concentrations of observed individuals. This was the case in the lower Loire valley, Rhône valley and Alps, for example. We also found different aggregation points: areas near the Gulf of Lion, the southern coast of the Bay of Biscay, the Atlantic coasts of Vendée, Normandy and Pas-de-Calais, along with some scattered data from England and the Rhine Valley. Few resightings were made in areas south of the natal colonies (Fig. 2).

The goodness-of-fit test for the AS model was rejected, indicating heterogeneity within the dataset (Table 1). A positive association test [43] revealed recapture heterogeneity among individuals (Test statistic: 7.645, *P* value: < 0.001) (see Additional file 1: Table S2). To account for this heterogeneity [43], we created two classes of individuals with different resighting probabilities (denoted h), similar to Peron et al. [44]. We assumed that individuals did not change their class during their lifetime (see Additional file 1: S3). To account for the remaining dispersion of the data, an overdispersion coefficient equal to 1.37 (c-hat of the WBWA component test; Table 1) was applied.

**Table 1:** The five components of the AS test for goodness of fit

	Test WBWA	Test 3G.SR	Test 3G.Sm	Test M.ITEC	Test M.LTEC	Global test
κ^2^	69.98	22.53	149.91	120.02	86.13	448.88
*df*	51	5	165	50	47	318
*p* value (κ^2^)	0.40	**< 0.01**	0.79	**< 0.01**	**< 0.01**	**< 0.01**
c-hat	1.37	4.50	0.91	2.40	1.83	1.41

*κ*^*2*^ the κ^2^ statistics X^2^ (i), *df* degrees of freedom, *c-hat* dispersion coefficient, significant values are indicated in bold

The general model, model I (Table 2), included survival φ dependent on age (a), season in interaction with year (t) and natal region (g), a transition probably ψ dependent on the zone of origin (f) and the zone of destination (to), age, the interaction between season and year-season and natal region, as well as a recapture probability p dependent on the site, the interaction between year-season, natal region and considering two groups of individuals with different recapture probabilities (h). This model is denoted: φ (a.t.g), ψ (f.to.a.t.g), p (h.f.t.g) where a dot(.) indicates the interaction between two effects.

**Table 2:** Twelve of the top models used in model selection procedures

Model	φ	ψ	P	#Par	Deviance	QAIC	QAICc	ΔAICc
**A**	**a(1,2).g(HM + C)**	**f.to.a(1,2).season.g(HM + C)**	**h.f.t**	**148**	**25,250.64**	**18,727.13**	**18,732.77**	**0**
B	a(1,2).g(HM + C) + t	f.to.a(1,2).season.g(HM + C)	h.f.t	169	25,199.23	18,731.60	18,738.97	6.20
C	a(1,2).g(HM + C).t	f.to.a(1,2).season.g(HM + C)	h.f.t	196	25,129.44	18,734.64	18,744.59	11.82
D	a(1,2).g(HM + C)	f.to.a(1,2).season.g(HM + C)	h.f(1,2).g(HM + C).t + f(3,4).t	192	25,145.09	18,738.08	18,747.61	14.85
E	a(1,2).g.t	f.to.a(1,2).season.g	h.f.t	249	25,084.18	18,807.62	18,823.76	90.99
F	a(1,2).g.t	f.to.a(1,2).season.g	h.f.t.g	339	24,862.26	18,825.64	18,855.86	123.1
G	a(1,2).g.t	f.to.a(1,2).season.g(CM + H)	h.f.t	222	25,367.14	18,960.16	18,972.95	240.18
H	a(1,2).t	f.to.a(1,2).season	h.f.t	143	25,831.49	19,141.10	19,146.37	413.6
**I**	**a(1,2).g.t**	**f.to.a(1,2).g.t**	**h.f.t.g**	**565**	**24,696.61**	**19,156.72**	**19,243.14**	**510.37**
J	a(1,2).t	f.to.a(1,2).t	h.f.t	351	25,720.54	19,476.12	19,508.57	775.80
K	a(1,2)	f.to.a(1,2)	h.f	23	28,163.51	20,603.31	20,603.45	1870.68
L	a(1,2)	f.to.a(1,2)	f	22	28,274.54	20,682.35	20,682.48	1949.71

The best performing model (Model A) and the general model (Model I) are indicated in bold. See Additional file 1: Table S4 for a complete list of all 42 models

*QAIC* Aikake Information Criterion, *QAICc* Aikake Information Criterion corrected for small sample, *#Par* number of parameters, *ΔAICc* difference of AICc between the models and the model A. φ = survival, ψ = transition, P = recapture, a = age with a(1) = individuals less than 6 months old and a(2) = individuals over 6 months old, f = original zone, to = zone of destination, t = season.year, g = location of birth (C = Camargue, H = Hyères, M = Marseille), h = two groups of individuals with different recapture probabilities

Model selection considered 42 alternative models based on this general model (see Additional file 1: Table S4) to compare the effects of different parameters. Subsequently, the model with the lowest AICc was selected (model A in Table 2). This model has 148 parameters and a ΔAICc > 2 compared to the second best model, a sufficiently large difference to use it alone for parameter estimation. This best performing model included age- and natal region- dependent survival. An effect of region of origin and age was also shown on movement, as well as a seasonal effect (pre and post-breeding periods). The probability of resighting depended on the resighting zone, and the year-season, i.e. a spatio-temporal effect. A model without capture heterogeneity (Model L) was tested and, as expected, was less efficient than models with capture heterogeneity, such as model K (Table 2). This confirms the presence of individual recapture heterogeneity in this dataset (see “Discussion” section for more details).

### Variation in movement with age

**Fig. 3:**
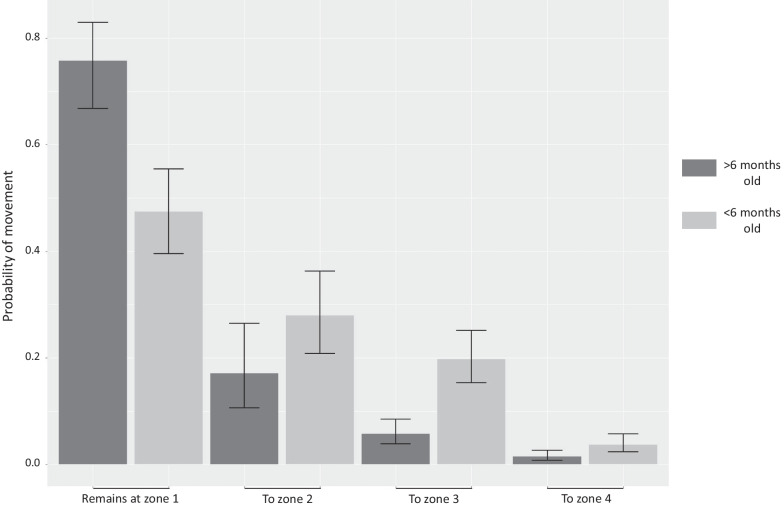
Age-dependent movement. Estimated post-breeding movement probabilities from the natal colony (+ 95% confidence interval) for individuals from Camargue colonies according to age. Individuals that remain at zone 1 are considered sedentary

Models that considered an age effect on movement performed better than those that did not—juveniles and immatures/adults therefore moved differently. It should be noted that only post-breeding movements could be compared by this model as all individuals entered into the same age class during the following pre-breeding period. It can be seen in Fig. 3 that older individuals from the Camargue were more likely to be sedentary in their natal zone (0.76 [0.66–0.83]) compared to juveniles (0.47 [0.40–0.55]). Individuals over 6 months old from Hyères and Marseille were also largely sedentary, with a probability of 0.99 [0.99–1] of remaining around the colony of origin. This is drastically different from juveniles from these regions which only had a 0.01[0.00–0.04] probability of staying. In general, juveniles moved to more distant areas, such as the Atlantic fringe, the North Sea and continental Europe, compared to older individuals.

### Variation in movements among natal regions

**Fig. 4:**
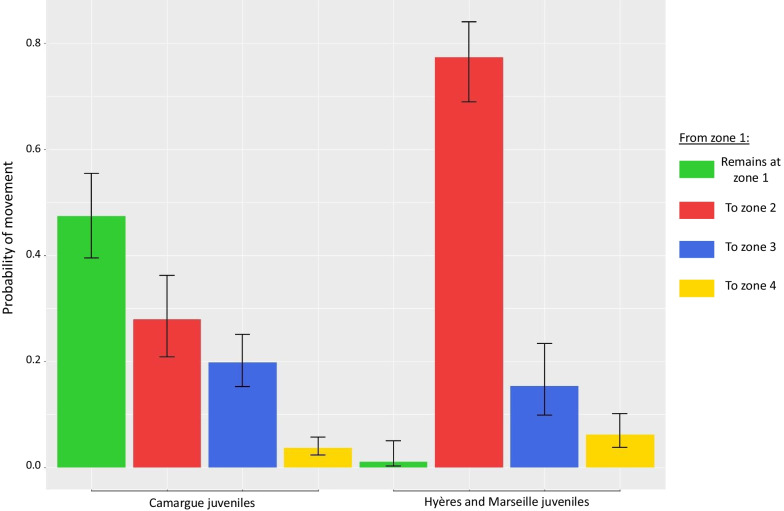
Colony-dependent movement. Estimated juvenile post-breeding movement probabilities (+ 95% confidence interval) according to the natal region. Here, the colonies from the Hyères and Marseille regions have been combined, as defined by the selected model (see “Results” section)

Models that considered an effect of natal region on movement performed better than those that did not, especially when the natal regions of Marseille and Hyères were combined (Table 2). This means that avian movements from colonies of Hyères and Marseille were similar, but differed from those of the Camargue. Figure 4 shows the transition probabilities of juveniles according to their natal region during the first 6 months of life. As outlined above, the probability for a Camargue juvenile to stay in zone 1, i.e., within 50 km of its natal colony, was much higher than that of a juvenile from colonies around Hyères and Marseille. The probability for juveniles from Hyères and Marseille to move to other regions of the Mediterranean basin (zone 2) were quite high (0.77 [0.68–0.84]), whereas their probabilities to move to more distant areas were lower: 0.15 [0.1–0.23] to zone 3 and 0.06 [0.04–0.1] to zone 4. Camargue juveniles that moved away from their natal colony tended to go to the northeast part of the Mediterranean basin (0.28 [0.21–0.36]) or along the Atlantic coast (0.20 [0.15–0.25]), with a very low probability of moving towards zone 4 (0.04 [0.02–0.06]). As outlined above, few immature/adult individuals from the colonies of Hyères/Marseille moved outside zone 1. Those from the Camargue had a higher probability to move, but most remained in the Mediterranean region (i.e., zone 2) (Fig. 3).

### Survival

According to the best model (Model A in Table 2), survival probability depended on age and natal region. After 6 months of age, survival probability was relatively constant. A time-effect on survival was not retained, as both model B (an additive effect of time) and model C (a multiplicative effect of time) were less efficient than model A. All possible associations between the three natal regions were tested and survival did not differ between Marseille and Hyères datasets. For immature/adult individuals, the probability of survival during a season was significantly lower in Marseille and Hyères 0.84 [0.80–0.86] compared to individuals in Camargue 0.89 [0.87–0.90]. In both regional groups, juveniles had lower survival probabilities than older individuals, but the difference was smaller in the Camargue. During the first 6 months after fledging, individuals from Camargue had a survival probability of 0.83 [0.72–0.92], i.e. much higher than for individuals originating from the Hyères/Marseille regions (0.43 [0.35–0.51]).

## Discussion

To the best of our knowledge, only two studies have described Yellow-legged gull movements using mark-recapture modeling, with one study focused on gulls of the *michahellis* subspecies from Croatia [25], and a second on the *lusitanius* subspecies from Spain [23]. Our capture-mark-recapture study therefore provides important additional results to evaluate the effect of age on movement in this species. Our results highlight once again the high mobility of juvenile gulls compared to more sedentary older birds. Interestingly, our work also indicates differences in movement patterns depending on the natal regions. Camargue juveniles were much more sedentary than juveniles from Hyères and Marseille, who almost all moved away from their natal colony zone during the non-breeding period. Among migrating individuals, the areas reached were always in Western Europe. For example, coastal areas of the Bay of Biscay were important, but more distant areas in Great Britain and the Netherlands were also sometimes reached. Our results partially contrast those for Croatian YLGs [26]. As for Croatian YLGs, the farthest areas reached by YLGs of southern France during migration were located further north. However, individuals from southern France had a tendency to move northwest, like populations of eastern Spain [33, 45], while those studied in Croatia moved more northeast, with only a slight overlap in the areas used by birds of the two groups in the lower Rhine valley.

Increased mobility in young birds is a frequent observation in seabirds and has been found in other gull species, such as the Lesser black-backed gulls (*L. fuscus*) [46–48], but also in other seabird groups (albatrosses [10–12], eiders [49] and cormorants [50]). However, the pattern is not systematic. For example, no effect of age on migration distance was found in herring gulls (*L. argentatus*) [51]; in this study, only migration departure and return dates varied with age. More widespread movements in juveniles could be an adaptation to avoid low food resources during the post-breeding period, shortages which may occur due to temporal changes in availability and/or increased competition around the breeding area. Older, more experienced, individuals are better competitors and should have acquired a repertoire of alternative foraging locations to avoid this problem. Adult gulls can also be under strong competition to secure high quality nest sites, limiting their ability to move too far from the breeding colony [27]. As juvenile birds do not reach sexual maturity until 4 years old, there is no initial constraint for them to remain locally. Indeed, early wide-scale movements allow young birds to prospect in order to find an optimal breeding area. Prospecting in colonies tends to be most intense at the time of fledgling when the overall quality of the local environment can be evaluated by local breeding success [52]. However, no studies to date have evaluated the role of juvenile prospecting in habitat selection in YLGs.

Among-natal region differences in movement behaviors could be linked to several non-mutually exclusive factors such as social transmission, where juveniles follow migrating adults [53, 54], or landscape features that facilitate or impede flying in specific directions [55]. It might also be associated with the quality of the local environment. Here, we used survival probabilities as an indicator of local conditions in the colony. We found lower survival estimates for both juveniles and immatures/adults coming from colonies in the Hyères and Marseille regions. This suggests that breeding conditions may have been more difficult in these areas, lowering the relative cost of migration/dispersion for these individuals [7, 56–58]. Regional differences in survival could be explained by resource variation around the breeding sites. For example, it has been shown that landfill closures can impact YLG population demographics via their effect on juvenile survival [59]. It is possible that there was a reduction in accessible garbage around Marseille/Hyères during the study period which forced individuals to migrate further, particularly if alternative food sources were not readily available. It is also possible that differences in migration strategies come from temporal differences in food availability. For example, the proximity of a colony to an landfill could ensure a reliable food source throughout the year [60] such that individuals do not need to migrate. Interestingly, near the Camargue, one of the largest open-air dumps in Europe, the Entressen landfill, was still in operation at the time of this study [61] and may explain why a large proportion of juveniles from this region were considered as sedentary by the model. If juveniles from Marseille and Hyères regions also used this dump for feeding, this would also explain why most young from these colonies were found to move to zone 2 (i.e., Entressen is more than 50 km from the natal colonies of Marseille/Hyères). The Entressen dump closed in 2010. It would thus be pertinent to investigate this hypothesis more fully, by comparing contemporary juvenile movements to the results of the present study. Interestingly, the diversity of resources available to foraging gulls is much higher in the Camargue compared to the Marseille/Hyères region, with more agricultural land and a more natural littoral zone. We therefore might expect that gull survival in the Camargue has remained relative stable over time, regardless of a reduction in available garbage. This is less likely to be the case for gulls living the in the Marseille/ Hyères region.

In addition to the potential impact of food resources on survival and migration probability, the proportion of migratory individuals within a region may also be conditioned by overall local breeding success, which depends in part on the quality of the local environment [62, 63]. Indeed, the studied colonies likely varied in quality in relation to factors such as population density, vegetation cover, predation, human disturbance, pollution, nest parasites or circulating pathogens. Interestingly, no effect of time was found on survival and/or movement in our study, suggesting that the potential impact of environmental stresses did not change during the study period. However, to detect this effect, a larger dataset of individuals than we considered here may be necessary. If the colonies of Marseille/Hyères represent lower quality breeding locations, we could expect a stronger decline in population size over time in these areas relative to the Camargue, both due to a reduction in natal recruitment and to lower emigration rates into the area (i.e., colonial seabirds are known to use conspecific reproductive success to select breeding habitat) [62]. Although population sizes have declined in the Mediterranean region [64], the role of local breeding habitat quality versus active management strategies, both to close open-air landfill sites and to reduce gull population sizes, cannot be disentangled. Future work will need to consider the role of such factors more carefully in order to better understand the origin of among-colony differences in survival and movement.

In our study, resighting data was divided into two 6-month time periods, post-breeding and pre-breeding, in order to obtain robust parameter estimates. However, this division limited our ability to examine survival and movement over shorter time intervals. Indeed, short-term movements have been observed in Herring gull adults, which have shorter wintering periods than immatures and juveniles [51]. For our dataset, dividing the data into shorter resighting periods, like a month, would have led to an overly complex model with little power to provide precise estimates. As our survival estimates are close to those obtained in other independent studies [23, 25, 32, 59, 65], we are confident that our results are generally robust. To evaluate this issue more completely, a mark-recapture dataset with more resighting data is required so that models can be run at shorter time intervals. Alternatively, survival and movement over short-time intervals will need to be measured directly from biologging data on adults and juveniles.

In our analyses, we observed recapture heterogeneity among Yellow-legged gull individuals which led us to include two classes of individuals in our models. The direct source of this heterogeneity is unknown, but likely arises from differences in individual behavior that alter resighting probability. For example, foraging behavior in YLGs can differ greatly both among individuals and among colonies [66]. Individual specialization on particular food resources has been previously observed, with some individuals feeding only at landfills, and others only at sea [67, 68]. The probability of resighting an individual is surely much higher for birds that use landfills because these zones are visited by ornithologists wanting to read rings. Few examples exist in the literature of Mark-Recapture datasets in which individual heterogeneity is corrected for directly in the model, adding a fundamental interest to our results [44].

The Yellow-legged gull populations studied here are relatively new since this species did not breed on the French coast prior to 1908. The development of centralized open-air landfills and trawling, in combination with high intrinsic vagility, probably played a major role in this expansion and in the strong increase in population densities seen during the last part of the twentieth century [36]. Indeed, widespread juvenile movements and partial migration may help these gulls adapt to novel environmental conditions [29]. However, movement and migration can also be costly. The energetic costs of movement and the exploration of unknown areas can lead to high direct mortality [5]. These movements can also influence exposure to parasites and pathogens, homogenizing their distribution in the environment [69, 70]. Young individuals, which move in greater proportion and to more distant locations, have a higher potential to expose themselves to novel parasites and pathogen and to disperse these agents at different spatial scales than adults [1]. These differences in movement may also create disparities in exposure to other types of environmental stresses such as pollutants that, like pathogens, can have direct consequences on seabird population dynamics [63, 71, 72]. As the presence of diverse environmental stressors can directly impact reproductive success, which in turn can motivate movements, a negative feedback loop may exist between these factors. The use of GPS tags to study the continuous movement of individuals of different ages and from different locations should enable us to obtain a more accurate view of the consequences of age specific movements for population and ecosystem dynamics.

## Conclusions

In this study, we found that Yellow-legged gull movements differed according to age and natal colony. These results provide valuable insights into the movement ecology of this species in the western part of its range. This study demonstrates the importance of studying individuals of different ages and from different colonies/locations when trying to understand migration strategies. Indeed, we show that movements can differ at a relatively small spatial scale, here between colonies only 50 km apart. Similarly, the movement patterns described here took into account heterogeneity in recapture probability, a bias rarely corrected for in studies of seabird movements. This bias is likely to be an important source of variation in other model systems, hindering robust parameter estimation. The method used to correct for capture heterogeneity in this study is thus an example that can inspire other studies facing heterogeneity in their data.

## Supplementary Information

**Additional file 1**. Contains additional tables including the heterogeneity test, the full model selection, a description of the different age class typologies tested, and the transition and event matrices of the model.

## Data Availability

The datasets generated and/or analysed during the current study are available in the Wedrop repository, (https://ftp.cx/dxg8cC) under the name: CMR_YLG_dataset.txt.

## Abbreviations

YLG: Yellow-legged gull
AICc: Aikake Information Criterion [corrected for small samples]
AS: Arnason-Schwarz model
