# Supplementary material for: Natal colony influences age-specific movement patterns of the Yellow-legged gull (Larus michahellis)
Source: Mov Ecol. 2023 Feb 11;11:11. doi: 10.1186/s40462-023-00375-4 (PMC9922451; doi:10.1186/s40462-023-00375-4)
Supplement: Supplementary file 1 — Additional file 1. Contains additional tables including the heterogeneity test, the full model selection, a description of the different age class typologies tested, and the transition and event matrices of the model. [file 40462_2023_375_MOESM1_ESM.docx]

Souc et al,

**Natal colony influences age-specific movement patterns of the Yellow-legged gull (Larus michahellis)**

**Additional file**

S1: The three age class typologies used during the model selection, with Oi = Occasion of capture number i, each of these occasions is for a period of six months, in column: Cohort which is the year of ringing just after birth, Ai : Age class number i.

a(1,2) : 2 Age classes with A1 during the first semester after fledging and A2 after the first semester.

| Cohort | O1 | O2 | O3 | O4 | O5 | O6 | O7 | O8 | O9 | … |
| --- | --- | --- | --- | --- | --- | --- | --- | --- | --- | --- |
| 1999 | A1 | A2 | A2 | A2 | A2 | A2 | A2 | A2 | A2 | … |
| 2000 | - | - | A1 | A2 | A2 | A2 | A2 | A2 | A2 | … |
| 2001 | - | - | - | - | A1 | A2 | A2 | A2 | A2 | … |
| 2002 | - | - | - | - | - | - | A1 | A2 | A2 | … |
| … |  |  |  |  |  |  |  |  |  | … |

a(1yo,+1yo) : 2 Ages class with A1 during the first year after fledging and A2 after the first year.

| Cohort | O1 | O2 | O3 | O4 | O5 | O6 | O7 | O8 | O9 | … |
| --- | --- | --- | --- | --- | --- | --- | --- | --- | --- | --- |
| 1999 | A1 | A1 | A2 | A2 | A2 | A2 | A2 | A2 | A2 | … |
| 2000 | - | - | A1 | A1 | A2 | A2 | A2 | A2 | A2 | … |
| 2001 | - | - | - | - | A1 | A1 | A2 | A2 | A2 | … |
| 2002 | - | - | - | - | - | - | A1 | A1 | A2 | … |
| … |  |  |  |  |  |  |  |  |  | … |

a(1yo,3yo,+3yo) : 3 Age class with A1 during the first year after fledging, A2 between the first year and the third year, and A3 after the third year.

| Cohort | O1 | O2 | O3 | O4 | O5 | O6 | O7 | O8 | O9 | … |
| --- | --- | --- | --- | --- | --- | --- | --- | --- | --- | --- |
| 1999 | A1 | A1 | A2 | A2 | A2 | A2 | A3 | A3 | A3 | … |
| 2000 | - | - | A1 | A1 | A2 | A2 | A2 | A2 | A3 | … |
| 2001 | - | - | - | - | A1 | A1 | A2 | A2 | A2 | … |
| 2002 | - | - | - | - | - | - | A1 | A1 | A2 | … |
| … |  |  |  |  |  |  |  |  |  | … |

Table S2: The test of heterogeneity (positive association) by occasion from 3 to 20 provided by U-CARE. The last row is the overall test making the sum of all the components associated to each occasion. N is the number of individuals used for the positive association test denoted **test statistic.** (Jeyam et al. 2018) When there is enough data, the test_statistic and its P_value are computed. When N<30 for occasions 19 and 20, a nonparametric permutation test is used.

| **Capture Occasion** | **Test statistics** | **N** | **P_value** |
| --- | --- | --- | --- |
| 3 | 4.6860 | 302 | <0.001 |
| 4 | 4.5660 | 281 | <0.001 |
| 5 | 6.1010 | 597 | <0.001 |
| 6 | 5.8360 | 450 | <0.001 |
| 7 | 5.3780 | 453 | <0.001 |
| 8 | 5.2770 | 361 | <0.001 |
| 9 | 5.3930 | 410 | <0.001 |
| 10 | 5.5270 | 344 | <0.001 |
| 11 | 4.8350 | 354 | <0.001 |
| 12 | 4.9210 | 289 | <0.001 |
| 13 | 5.4480 | 201 | <0.001 |
| 14 | 5.1430 | 158 | <0.001 |
| 15 | 4.8900 | 124 | <0.001 |
| 16 | 4.9640 | 101 | <0.001 |
| 17 | 3.7320 | 53 | <0.001 |
| 18 | 3.7500 | 44 | <0.001 |
| 19 | Na | 19 | 0.0960 |
| 20 | Na | 14 | 0.1500 |
| Global | 7.6450 | 986 | <0.001 |

S3: Transition and event matrices of the model with four geographic states and two capture groups in the framework of multi-event models (Pradel 2009).

The states are as follows:

Si: individual present in site (or region) i with high catchability

Si*: individual present on site i with low catchability

Death

The events are the following:

NS: Not seen

Ei: individual seen on site i

Transitions are modeled as the product of the survival matrix Φ and the movement matrix Ψ.

Event are modeled as the product of the capture matrix P and the heterogeneity matrix C that include a capturability coefficient c for weakly capturable individual

1. – Survival Matrix

|  | **S1** | **S2** | **S3** | **S4** | **S1*** | **S2*** | **S3*** | **S4*** | **Dead** |
| --- | --- | --- | --- | --- | --- | --- | --- | --- | --- |
| **S1** | Φ1 | 0 | 0 | 0 | 0 | 0 | 0 | 0 | 1- Φ1 |
| **S2** | 0 | Φ2 | 0 | 0 | 0 | 0 | 0 | 0 | 1- Φ2 |
| **S3** | 0 | 0 | Φ3 | 0 | 0 | 0 | 0 | 0 | 1- Φ3 |
| **S4** | 0 | 0 | 0 | Φ4 | 0 | 0 | 0 | 0 | 1- Φ4 |
| **S1*** | 0 | 0 | 0 | 0 | Φ1 | 0 | 0 | 0 | 1- Φ1 |
| **S2*** | 0 | 0 | 0 | 0 | 0 | Φ2 | 0 | 0 | 1- Φ2 |
| **S3*** | 0 | 0 | 0 | 0 | 0 | 0 | Φ3 | 0 | 1- Φ3 |
| **S4*** | 0 | 0 | 0 | 0 | 0 | 0 | 0 | Φ4 | 1- Φ4 |
| **Dead** | 0 | 0 | 0 | 0 | 0 | 0 | 0 | 0 | 1 |

(b) – Movement Matrix

|  | **S1** | **S2** | **S3** | **S4** | **S1*** | **S2*** | **S3*** | **S4*** | **Dead** |
| --- | --- | --- | --- | --- | --- | --- | --- | --- | --- |
| **S1** | ψ11 | Ψ12 | Ψ13 | Ψ14 | 0 | 0 | 0 | 0 | 0 |
| **S2** | Ψ21 | ψ22 | Ψ23 | Ψ24 | 0 | 0 | 0 | 0 | 0 |
| **S3** | Ψ31 | Ψ32 | ψ33 | Ψ34 | 0 | 0 | 0 | 0 | 0 |
| **S4** | Ψ41 | Ψ42 | Ψ43 | ψ44 | 0 | 0 | 0 | 0 | 0 |
| **S1*** | 0 | 0 | 0 | 0 | ψ11 | Ψ12 | Ψ13 | Ψ14 | 0 |
| **S2*** | 0 | 0 | 0 | 0 | Ψ21 | ψ22 | Ψ23 | Ψ24 | 0 |
| **S3*** | 0 | 0 | 0 | 0 | Ψ31 | Ψ32 | ψ33 | Ψ34 | 0 |
| **S4*** | 0 | 0 | 0 | 0 | Ψ41 | Ψ42 | Ψ43 | ψ44 | 0 |
| **Dead** | 0 | 0 | 0 | 0 | 0 | 0 | 0 | 0 | 1 |

ψ11= 1- ψ12- ψ13- ψ14

ψ22=1- ψ21- ψ23- ψ24

ψ33=1- ψ31- ψ31 ψ34

ψ44=1- ψ41- ψ42- ψ43

(c) – Capture Matrix

|  | **NS** | **E1** | **E2** | **E3** | **E4** | **E1*** | **E2*** | **E3*** | **E4*** |
| --- | --- | --- | --- | --- | --- | --- | --- | --- | --- |
| **S1** | 1-P | P | 0 | 0 | 0 | 0 | 0 | 0 | 0 |
| **S2** | 1-P | 0 | P | 0 | 0 | 0 | 0 | 0 | 0 |
| **S3** | 1-P | 0 | 0 | P | 0 | 0 | 0 | 0 | 0 |
| **S4** | 1-P | 0 | 0 | 0 | P | 0 | 0 | 0 | 0 |
| **S1*** | 1-P | 0 | 0 | 0 | 0 | P | 0 | 0 | 0 |
| **S2*** | 1-P | 0 | 0 | 0 | 0 | 0 | P | 0 | 0 |
| **S3*** | 1-P | 0 | 0 | 0 | 0 | 0 | 0 | P | 0 |
| **S4*** | 1-P | 0 | 0 | 0 | 0 | 0 | 0 | 0 | P |
| **Dead** | 1-P | 0 | 0 | 0 | 0 | 0 | 0 | 0 | 0 |

(d) – The coefficient C between the rate of capture is implemented in the Heterogeneity Matrix

|  | **NS** | **E1** | **E2** | **E3** | **E4** |
| --- | --- | --- | --- | --- | --- |
| **S1** | 1 | 1 | 0 | 0 | 0 |
| **S2** | 0 | 0 | 1 | 0 | 0 |
| **S3** | 0 | 0 | 0 | 1 | 0 |
| **S4** | 0 | 0 | 0 | 0 | 1 |
| **S1*** | 1-C | C | 0 | 0 | 0 |
| **S2*** | 1-C | 0 | C | 0 | 0 |
| **S3*** | 1-C | 0 | 0 | C | 0 |
| **S4*** | 1-C | 0 | 0 | 0 | C |

Table S4: Models based on model selection procedures, where φ=survival, ψ=transition, p=capture, a=age with a(1,2), a(1yo,+1yo), a(1yo,3yo,+3yo), the difference between this three ages class are specified in the appendix 4, f=original site, to=site of destination, t=season*year, g=region of birth (C=Camargue, H=Hyères, M=Marseille), h=taking into account two groups of individuals with different recapture probabilities.

| Φ | ψ | P | **#Par*** | **Deviance** | **QAIC*** | **QAICc*** | **ΔAICc*** |
| --- | --- | --- | --- | --- | --- | --- | --- |
| a(1,2).g(HM+C) | f.to.a(1,2).season.g(HM+C) | h.f.t | 148 | 25250.64 | 18727.13 | 18732.77 | 0 |
| a(1,2).g(HM+C)+t | f.to.a(1,2).season.g(HM+C) | h.f.t | 169 | 25199.23 | 18731.60 | 18738.97 | 6.20 |
| a(1,2).g(C)+a(1,2).g(HM).t | f.to.a(1,2).season.g(HM+C) | h.f.t | 173 | 25192.43 | 18734.64 | 18742.36 | 9.59 |
| a(1yo,+1yo).g(HM+C)+t | f.to.a(1,2).season.g(HM+C) | h.f.t | 169 | 25204.48 | 18735.43 | 18742.80 | 10.03 |
| a(1,2).g(HM+C).t | f.to.a(1,2).season.g(HM+C) | h.f.t | 196 | 25129.44 | 18734.65 | 18744.59 | 11.82 |
| a(1,2).g(HM+C) | f.to.a(1,2).season.g(HM+C) | h.f(1,2).g(HM+C).t+h.f(3,4).t | 192 | 25145.09 | 18738.08 | 18747.61 | 14.85 |
| a(1,2).g+t | f.to.a(1,2).season.g | h.f.t | 198 | 25165.96 | 18765.31 | 18775.46 | 42.69 |
| a(1,2).g(C)+a(1,2).g(HM).t | f.to.a(1,2).season.g | h.f.t | 200 | 25163.21 | 18767.30 | 18777.66 | 44.89 |
| a(1,2).t.g(HM+C) | f.to.a(1,2).season.g | h.f.t | 223 | 25100.21 | 18767.32 | 18780.23 | 47.46 |
| a(1,2).g(HM+C) | f.to.a(1,2).season.g(HM+C) | h.f.t.g(HM+C) | 272 | 24988.67 | 18783.90 | 18803.20 | 70.44 |
| age(1yo,+1yo).g(HM+C)+t | f.to.age(1yo,+1yo).season.g(HM+C) | h.f.t | 193 | 25207.47 | 18785.62 | 18795.25 | 62.48 |
| a(1,2).t+g | f.to.a(1,2).season.g | h.f.t | 200 | 25190.79 | 18787.43 | 18797.79 | 65.02 |
| a(1,2).g(HM+C) | f.to.a(1,2).season.g(HM+C) | h.f.t.g(HM+C) | 272 | 24988.67 | 18783.90 | 18803.20 | 70.44 |
| a(1,2).g(C)+a(1,2).t.g(H,M) | f.to.a(1,2).season.g | f.t | 226 | 25148.66 | 18808.68 | 18821.94 | 89.17 |
| a(1,2).g.t | f.to.a(1,2).season.g | h.f.t | 249 | 25084.18 | 18807.62 | 18823.76 | 90.99 |
| a(1,2).t.g(HC+M) | f.to.a(1,2).season.g | h.f.t | 223 | 25174.97 | 18821.89 | 18834.79 | 102.03 |
| a(1,2).g.t | f.to.age(1).g+f.to.age(2).season | h.f.t | 201 | 25243.55 | 18827.95 | 18838.41 | 105.64 |
| a(1,2).g.t | f.to.a(1,2).season.g(HC+M) | h.f.t | 222 | 25190.41 | 18831.16 | 18843.94 | 111.18 |
| a(1yo,3yo,+3yo).t.g(HM+C) | f.to.age(1yo,3yo,+3yo).season.g(HM+C) | h.f.t | 243 | 25141.42 | 18837.40 | 18852.76 | 119.99 |
| a(1,2).g.t | f.to.a(1,2).season.g | h.f.t.g | 339 | 24862.26 | 18825.64 | 18855.86 | 123.10 |
| a(1,2).g(HM+C) | f.to.a(1,2).season.g(HM+C) | h.f.t.g | 300 | 24979.73 | 18833.38 | 18856.94 | 124.17 |
| a(1,2).g.t | f.to.a(1,2).season.g | h.f(1).t.g+h.f(2,3,4).t | 289 | 25013.47 | 18836.00 | 18857.84 | 125.07 |
| a(1,2).t.g(MC+H) | f.to.a(1,2).season.g | h.f.t | 223 | 25326.29 | 18932.34 | 18945.25 | 212.48 |
| a(1,2).g.t | f.to.a(1,2).season | h.f.t | 240 | 25283.22 | 18934.91 | 18949.88 | 217.11 |
| a(1,2).g.t | f.to.a(1,2).season.g(MC+H) | h.f.t | 222 | 25367.14 | 18960.16 | 18972.95 | 240.18 |
| a(1,2).g.t | f.to.a(1,2).season | h.f.t | 195 | 25450.59 | 18967.07 | 18976.91 | 244.14 |
| a(1,2).g(HM+C) | f.to.a(1,2).season.g(HM+C) | h.f+t | 85 | 25891.17 | 19068.67 | 19070.52 | 337.75 |
| a(1,2).g.t | f.to.season | h.f.t | 192 | 25654.63 | 19110.01 | 19119.54 | 386.77 |
| a(1,2).t | f.to.a(1,2).season | h.f.t | 143 | 25831.49 | 19141.10 | 19146.37 | 413.60 |
| t | f.to.a(1,2).season | h.f.t | 138 | 25854.62 | 19147.99 | 19152.89 | 420.12 |
| a(1,2) | f.to.a(1,2).season | h.f.t | 119 | 25911.27 | 19151.33 | 19154.97 | 422.21 |
| a(1,2).g.t | f.to.a(1,2).g,t | h.f.g.t | 565 | 24696.61 | 19156.72 | 19243.14 | 510.37 |
| a(1,2).t | f.to.a(1,2).t | h.f.t | 351 | 25720.54 | 19476.12 | 19508.57 | 775.80 |
| a(1,2) | f.to.a(1,2).t | h.f.t | 331 | 25786.03 | 19483.92 | 19512.70 | 779.94 |
| a(1,2).g(HM+C) | f.to.a(1,2).season.g(HM+C) | h.f(1,2).g(HM+C)+h.f(3,4) | 66 | 27018.33 | 19853.41 | 19854.53 | 1121.76 |
| t | f.to.a(1,2).season | h.f | 55 | 27293.34 | 20032.15 | 20032.93 | 1300.16 |
| a(1,2).g | f.to.a(1,2).season | h.f | 39 | 27371.33 | 20057.08 | 20057.47 | 1324.70 |
| a(1,2) | f.to.a(1,2).season | h.f.season | 35 | 27725.47 | 20307.57 | 20307.89 | 1575.12 |
| a(1,2) | f.to.a(1,2) | h.f | 23 | 28163.51 | 20603.31 | 20603.45 | 1870.68 |
| h.a(1,2) | f.to.a(1,2) | h.f | 25 | 28189.50 | 20626.28 | 20626.44 | 1893.68 |
| a(1,2) | f.to.a(1,2) | f | 22 | 28274.54 | 20682.35 | 20682.48 | 1949.71 |
| h.a(1,2) | f.to.a(1,2) | f | 23 | 28274.54 | 20684.35 | 20684.49 | 1951.72 |
| a(1,2) | f.to.a(1,2).season | h.f | 35 | 34933.70 | 25569.05 | 25569.37 | 6836.61 |

*QAIC = Aikake Information Criterion, QAICc = Aikake Information Criterion corrected for small sample #Par=number of parameters,* *Δ AICc* ***=*** *difference of AICc between the models and the model A.*
